# Automated Classification of Maxillary Sinus Ostium Patency Using a ConvNeXt-Tiny + DeiT Gated MLP-Based Hybrid Deep Learning Model: A Retrospective CBCT Study

**DOI:** 10.3390/diagnostics16101512

**Published:** 2026-05-16

**Authors:** Furkan Talo, Nurullah Duger, Emre Aslan, Muhammed Yildirim, Mahmut Kaya, Ahmet Bedri Ozer, Tuba Talo Yildirim

**Affiliations:** 1 Department of Computer Engineering, Firat University, Elazig 23119, Türkiye; ftalo@firat.edu.tr; 2 Department of Periodontology, Faculty of Dentistry, Firat University, Elazig 23119, Türkiye; nduger@firat.edu.tr (N.D.); e_arslan@firat.edu.tr (E.A.); taloyildirim@firat.edu.tr (T.T.Y.); 3 Department of Artificial Intelligence and Data Engineering, Firat University, Elazig 23119, Türkiye; muhammedyildirim@firat.edu.tr (M.Y.); mahmutkaya@firat.edu.tr (M.K.)

**Keywords:** artificial intelligence, CNN, deep learning, ostium, ViT

## Abstract

**Background/Objectives**: The patency and anatomical location of the maxillary sinus ostium are critical for preventing postoperative complications in dental implant planning and sinus lift surgeries in the posterior maxilla. Narrowing or obstruction of the ostium carries risks, including the development of acute/chronic sinusitis and bone graft failure after surgery. These risks must be carefully evaluated using preoperative radiographic images. It is time-consuming for physicians to manually perform this process, and details are overlooked due to a lack of clinical experience, which can increase surgical risks. **Methods**: This study aims to overcome these clinical challenges and improve the reliability of radiographic evaluation. In this study, a hybrid deep learning model is proposed for the automatic detection of the maxillary sinus ostium. The proposed model combines the local feature extraction power of CNN-based models with the global context modeling capabilities of transformer-based models, creating an effective model. Additionally, the gated fusion technique efficiently combines features from various designs, significantly enhancing classification performance. **Results**: The proposed model was compared with six different ViT and CNN architectures established in the literature. While the highest test accuracy among pre-trained models was 89.36%, the proposed hybrid model achieved 95.03%, demonstrating strong clinical diagnostic performance. **Conclusions**: Based on the performance metrics obtained, we believe the proposed model can be used to determine the patency of the maxillary sinus ostium. This will lighten the workload for specialists and minimize traditional errors.

## 1. Introduction

The maxillary sinus is a pyramid-shaped structure approximately 2.5 cm wide, 3.75 cm long, and 3 cm deep and is the largest of the paranasal sinuses [1]. This structure is lined with the sinus membrane, also known as the Schneiderian membrane. This membrane is connected to the middle meatus of the nose via the ostium located at the top of the medial wall of the sinus. The ostium is responsible for the drainage and ventilation of the sinus [2]. Anatomical variations and dental factors can obstruct the maxillary sinus ostium, hindering sinus drainage and ventilation. This increases susceptibility to infectious diseases, primarily sinusitis [3,4].

The incidence of sinusitis in sinus lift procedures has been reported to range from 4.2% to 8.4% [5,6,7]. Elevation of the Schneiderian membrane during sinus lift operations can cause thickening and an inflammatory response in the sinus mucosa [8]. Regression of these inflammatory changes is possible with an effective mucociliary clearance mechanism and adequate drainage to the nasal cavity via the sinus ostium; however, edema, mucosal trauma, or anatomical changes that develop after sinus lift surgery can disrupt this physiological process and negatively affect healing. The risk of developing postoperative sinusitis is generally associated with inflammatory edema in the sinus, displacement of graft material due to membrane perforation, or narrowing or complete occlusion of the ostium due to pre-existing chronic sinusitis [9]. Therefore, checking the osteomeatal complex and ostium patency during preoperative evaluation is important for better preparation and success in patients undergoing sinus lifts [10].

Traditional radiographic assessments of the maxillary sinus only provide two-dimensional images, which are often insufficient for accurate evaluation. Therefore, cone-beam computed tomography (CBCT) is recommended for more detailed imaging due to its superior spatial resolution [11]. Today, CBCT has become a widely used and preferred imaging method in dentistry for evaluating maxillary sinus anatomy and pathology due to its low radiation dose, high spatial resolution, and three-dimensional imaging capability [12]. The assessment of the ostium as “open” or “closed” involves significant subjectivity among clinicians. Inter-observer agreement in assessing ostium patency among radiologists and surgeons has been shown to be as low as 0.399 in some studies [13]. This highlights the need for objective decision-support systems that minimize experience-based errors.

In recent years, artificial intelligence (AI) techniques, especially deep learning (DL) algorithms, have made significant progress in medical image analysis [14,15,16]. Convolutional neural network (CNN)- and Vision Transformer (ViT)-based models show promising results in maxillary sinus segmentation and pathology detection [17,18]. AI-based studies concerning the ostium are quite limited in the literature. One of the few studies on this subject is the study by Shetty et al., who used the ResNet101V2 model for accessory ostium (AMO) detection in CBCT images and achieved 81% accuracy. They used a limited number of CNN models in their study [19]. Testing the model’s accuracy with various other models and using a hybrid model can improve accuracy. Nevertheless, the study by Shetty et al. is an important piece of evidence showing that artificial intelligence can learn ostium structures on CBCT images [19].

The aim of this study was to develop an objective, highly accurate, AI-based hybrid deep learning model to assist clinicians in evaluating maxillary sinus ostium patency. The model aims to automatically classify ostium status (open/closed) in preoperative CBCT images, contributing to safer surgical planning and the prevention of postoperative sinusitis complications. The proposed hybrid model achieved an accuracy of 95.03%.

## 2. Materials and Methods

### 2.1. Data Collection and Ethical Approval

The Firat University Non-Interventional Ethics Committee (FUGOEK) approved this retrospective study (No. 2025-17-40), and it was carried out in compliance with the Helsinki Declaration. A total of 500 CBCT images from patients who visited Firat University’s Faculty of Dentistry between January 2019 and September 2025 were analyzed. A ProMax 3D Mid (Planmeca, Helsinki, Finland) model tomography device was used to obtain CBCT images using a standard acquisition technique at 90 kVp and 8 mA with an exposure period of 8–9 s. The voxel size of the images was 0.4 mm. Three professional doctors used Romexis Viewer 4.4.3 (Planmeca, Helsinki, Finland) software to assess the images without any preprocessing, in accordance with the manufacturer’s recommendations. Based on whether the ostium was open or closed, a dataset comprising 704 PNG images—477 open and 227 closed—was produced and divided into two groups. Patients who had previously provided written informed consent for the anonymous use of their records for scientific purposes provided all of the radiography data used in this investigation, which was collected retrospectively. This consent was acquired during their first visit to the clinic. As a result, no further informed consent was needed. To avoid identifying the patients, the data were anonymized.

### 2.2. Inclusion-Exclusion Criteria and Dataset

Inclusion criteria included CBCT radiographic images of patients demonstrating a clearly identifiable and measurable maxillary sinus ostium in the upper jaw region.

There were no restrictions regarding patient age, gender, or clinician. Exclusion criteria included low-quality images due to exposure errors, artifacts, overlay, or distortion.

### 2.3. Manual Classification and Standardization

Manual classification of the ostium as open or closed was performed independently by three experts in the field of periodontology. Forty CBCT sections (Open Ostium [*n* = 20] and Closed Ostium [*n* = 20]) were chosen, and manual evaluations were repeated two weeks later in order to examine inter-observer and intra-observer consistency. The reliability of the measurements was evaluated using intraclass correlation coefficients (ICCs) to assess inter- and intra-observer consistency. Excellent intra-observer reliability was demonstrated by ICC values of 0.9418 and 0.9321, while strong inter-observer reliability was demonstrated by ICC values of 0.9315 and 0.9251. The high reliability of evaluations conducted over time and by many operators attests to the precision and consistency of the manual classification process.

**Figure 1:**
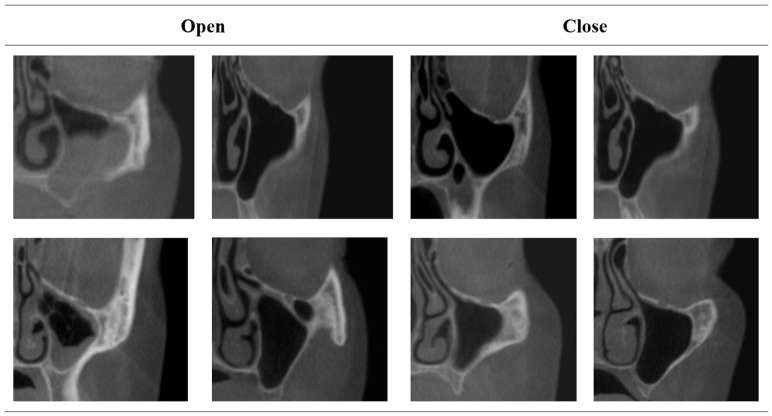
Sample images from the dataset.

The dataset contained 704 images showing both the upper and lower jaws. There were 477 images in the “open” class and 227 in the “closed” class. Sample images from the dataset are presented in Figure 1.

### 2.4. Proposed Model

**Figure 2:**
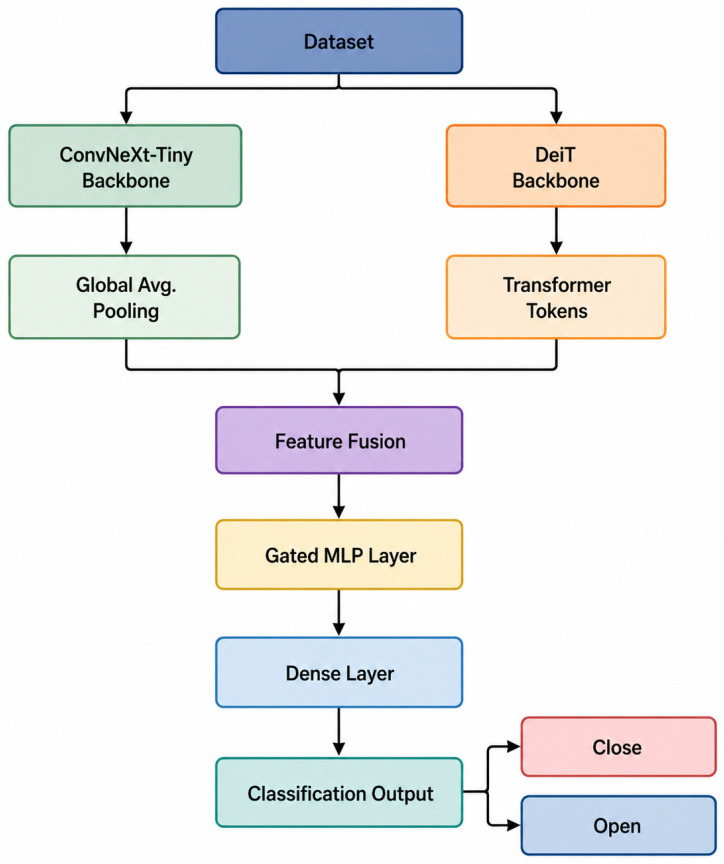
Flowchart of the proposed model for determining the maxillary sinus ostium.

In this paper, a hybrid model is proposed to automatically detect the maxillary sinus and classify whether the maxillary sinus ostium is open or closed. The developed model is a hybrid of ConvNeXt and DeiT architectures. The ConvNeXt architecture was chosen for its performance in local feature extraction with convolution-based networks, while DeiT was chosen to leverage transformer-based models’ ability to learn global contextual relationships. The model developed for maxillary sinus detection consists of three stages: feature extraction, feature fusion, and a classification head. The flowchart of the proposed model is presented in Figure 2.

As shown in Figure 2, the proposed model uses ConvNeXt-Tiny and DeiT architectures as backbones. The ConvNeXt-Tiny architecture efficiently learns local spatial patterns in images using a convolution-based structure. Feature maps obtained from the model are converted into a one-dimensional feature vector using global average pooling. On the other hand, the DeiT model learns long-range correlations and global contextual information within the image using a transformer-based architecture. Token representations obtained from the DeiT model are similarly converted into feature vectors. The feature vectors obtained from these two models are then transferred to the fusion layer. In the proposed model, feature fusion was performed using a gated mechanism. This mechanism dynamically regulates the information flow by learning the importance of features obtained from the ConvNeXt-Tiny and DeiT models. Thus, the model can emphasize more distinctive features while suppressing less important ones. In other words, the gated fusion mechanism takes feature vectors from the ConvNeXt-Tiny and DeiT models as input and adjusts the contribution of each feature using learnable gate functions. In this way, the proposed hybrid model balances the information from the ConvNeXt-Tiny and DeiT backbone models. This suppresses unnecessary features while creating a stronger combined representation. Finally, the combined feature vector obtained from the fusion stage is transferred to a multilayer perceptron (MLP)-based classification header. These layers generate the final class prediction using linear transformations and nonlinear activation functions. The final layer of the model calculates class probabilities using softmax activation. In this study, when selecting the backbone, the results of ResNet50 [20], DenseNet121 [21], ConvNeXt-Tiny [22], ViT-B/16 [23], DeiT [24], and Swin [25] models were evaluated, and the CNN and ViT, which achieved the highest test accuracy among these models, were selected. Therefore, the ConvNeXt-Tiny and DeiT models were used as the basis for the proposed model for determining the maxillary sinus ostium.

### 2.5. Train Strategy and Performance Measurement Metrics

A hybrid model was developed to automatically detect the maxillary sinus using cone-beam computed tomography images and to classify whether the maxillary sinus ostium is open or closed. The developed model was compared with 6 different pre-trained CNN and ViT architectures established in the literature. In this stage, different evaluation metrics, primarily confusion matrices, were used. In selecting these metrics, the macro-averaging technique was used to ensure balanced and unbiased evaluation across classes. In the macro-averaging method, each performance metric is first calculated separately for each class using the confusion matrix. Then, the arithmetic mean of the obtained values is taken to obtain the final result. This approach enables each class to contribute equally to the evaluation, regardless of the class distribution in the dataset. Accordingly, precision (Pre.), recall, and negative predictive value (NPV) were calculated using macro-averaging. The accuracy (Acc.) value was calculated as the ratio of the number of correctly classified samples to the total number of samples [26,27]. In addition, the Matthews correlation coefficient (MCC) [28], a balanced performance metric that considers all components of the confusion matrix, was included in the performance measurement metrics. These metrics assessed the models’ performance in detecting the maxillary sinus comprehensively and in a balanced way across both classes.

The dataset was first divided into training and test sets in an 80–20% ratio. Then, 10% of the training data was allocated for validation. As a result, the data was split into 72% training, 8% validation, and 20% test. A stratified sampling strategy was used to maintain class distribution across all subsets. The experiments on the pre-trained models, ablation studies, and the proposed model were conducted in the Google Colaboratory environment using an NVIDIA Tesla T4 GPU.

## 3. Result

A hybrid model was developed to automatically detect the maxillary sinus and determine whether its ostium is open or closed. This section examines the results and confusion matrices for pre-trained CNN and ViT models to compare the performance of the hybrid model and then presents ablation studies. Performance evaluation metrics obtained from the pre-trained models are presented in Table 1.

Table 1 shows that the two models with the highest test accuracy values are DenseNet121 and ConvNeXt-Tiny. These two models stood out as the most successful models for automatically detecting the maxillary sinus ostium from cone-beam computed tomography images, with a test accuracy rate of 89.36%. DenseNet121 and ConvNeXt-Tiny were followed by DeiT with 87.94% and ResNet50 and Swin with 86.52%. Among the pre-trained models used in the study, the least successful model at automatically detecting the maxillary sinus ostium was ViT-B/16, achieving 83.68%.

**Figure 3:**
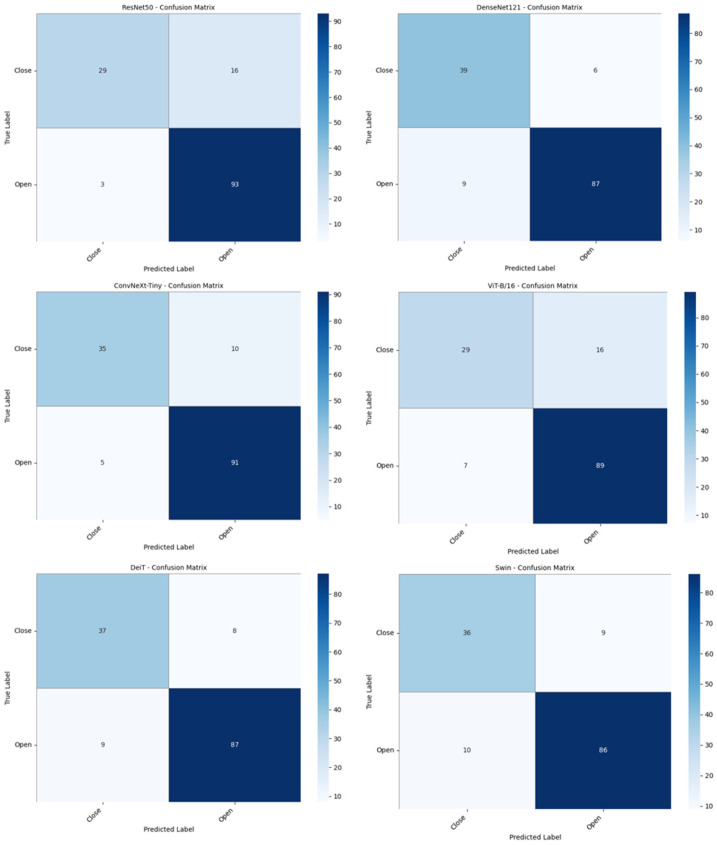
Confusion matrix of pre-trained models.

The confusion matrices obtained from the pre-trained models that automatically detected the maxillary sinus ostium using cone-beam computed tomography images are presented in Figure 3.

As shown in Figure 3, the confusion matrices for the CNN- and ViT-based models demonstrate that the DenseNet121 and ConvNeXt-Tiny models made the fewest errors. These models incorrectly predicted 15 images in the test dataset. ViT-B/16 incorrectly predicted 23 test images, ResNet50 19, Swin 19, and DeiT 17. In this study, a hybrid model was developed to automatically detect the maxillary sinus and classify whether its ostium is open or closed. The developed model was first compared with pre-trained CNN and ViT architectures. Then, the performance measurement metrics of the proposed model were calculated, and these metrics, along with ablation studies, are presented in Table 2.

Table 1 and Table 2 show that the ConvNeXt-Tiny and DeiT models, used as the baselines in the proposed model, achieved test accuracy of 89.36% and 87.94%, respectively. The ConvNeXt-Tiny + DeiT structure, however, reached an accuracy of 90.07%. This increase confirms that the CNN and Transformer representations offer complementary features. However, a significant change was observed in the recall value. While the proposed dual structure increased overall accuracy in this step, it remained limited in its capacity to capture complex samples. In the proposed model, the accuracy value increased to 95.03%. Furthermore, a noticeable increase was observed in other performance metrics. The reduction in the error rate to 4.96% also supports this. It appears that the gated mechanism highlights more distinctive representations by dynamically weighting the features from ConvNeXt and DeiT.

**Figure 4:**
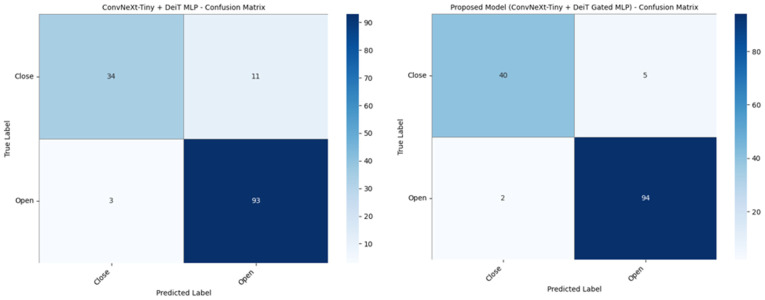
Confusion matrix of ConvNeXt-Tiny + DeiT MLP and the proposed model (ConvNeXt-Tiny + DeiT Gated MLP).

In conclusion, the proposed hybrid model demonstrates the highest classification performance among all models. Specifically, the 95.03% accuracy and 89.67 MCC value indicate that the proposed model exhibits very strong performance in detecting the maxillary sinus regarding both accuracy and balanced classification. Confusion matrices for ConvNeXt-Tiny + DeiT MLP and the proposed model (ConvNeXt-Tiny + DeiT Gated MLP) are presented in Figure 4.

**Figure 5:**
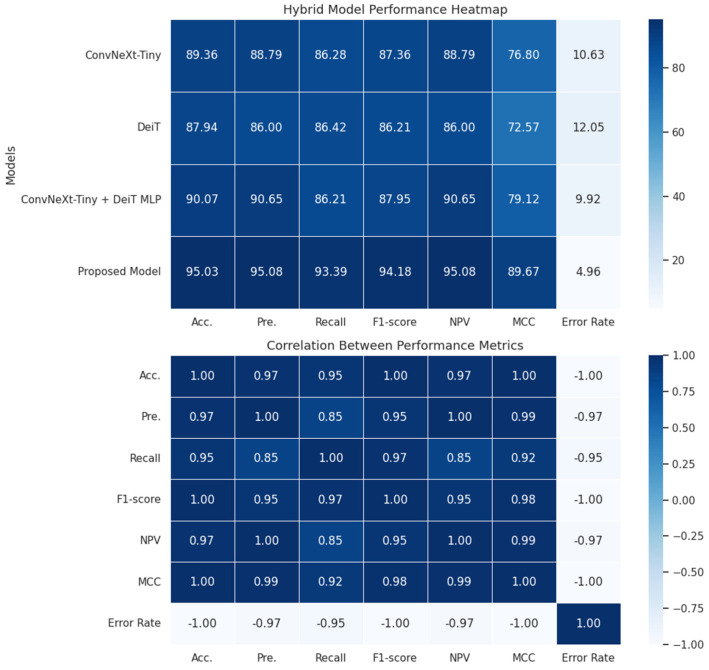
Performance heatmap and metric correlation analysis for the hybrid and proposed models.

The proposed model uses CNN-based ConvNeXt-Tiny and ViT-based DeiT models as baselines. Figure 3 shows that the ConvNeXt-Tiny model incorrectly predicted 15 images in the test dataset. The ViT-based DeiT model incorrectly predicted 17 images. In the proposed model, during the ConvNeXt-Tiny + DeiT MLP step—another ablation step—14 test images were incorrectly predicted, as shown in Figure 4. The number of incorrectly predicted images in the proposed model was 7. The performance heatmap and metric correlation analysis for the ablation models and the proposed model are presented in Figure 5.

**Figure 6:**
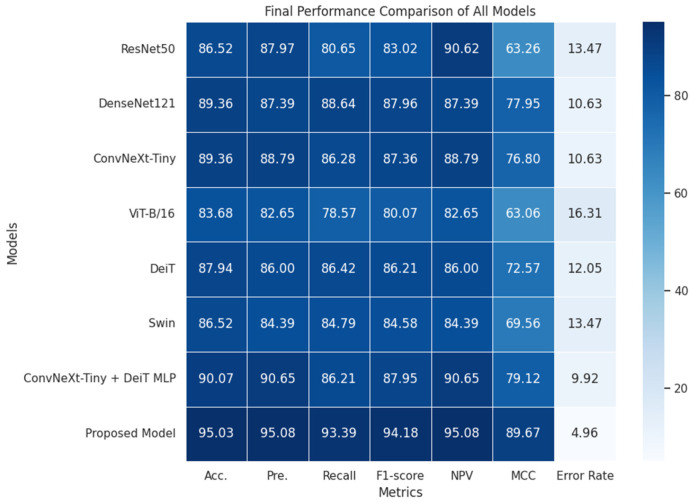
Comparison of performance metrics of all models.

Figure 5 shows that combining the features of the ConvNeXt-Tiny and DeiT models improves classification performance. Specifically, the ConvNeXt-Tiny + DeiT MLP model achieved higher accuracy and MCC than single-backbone models, indicating that combining features from different architectures enhances the model’s representational power. Furthermore, the superior performance of the proposed gated MLP-based architecture over the classical MLP fusion method indicates that more effective control of information flow between features improves model performance. The correlation analysis presented in the second heatmap shows the relationships between performance metrics. The analysis results reveal strong positive correlations between accuracy, F1-score, and MCC metrics. However, a strong negative correlation is observed between the error rate and other performance metrics. These results indicate that the evaluation metrics are consistent with one another and that the model’s performance is reliably assessed. A graph showing the metric-based performance of all models is presented in Figure 6.

Figure 6 shows that the best performance is obtained with the proposed model. Furthermore, the proposed model has the lowest error rate.

## 4. Discussion

Sinus floor elevation surgery is a frequently performed surgical procedure for implant treatment in atrophic posterior maxillary tissue, but it can lead to inflammatory reactions and mucosal thickening due to traumatic elevation of the Schneiderian membrane. These inflammatory changes can obstruct the ostium, the primary drainage pathway of the maxillary sinus into the nasal cavity, disrupting physiological mucociliary clearance [29,30]. Obstruction of drainage can result in mucosal edema and bacterial sinus infections. This can predispose the patient to acute or chronic maxillary sinusitis, leading to loss of the placed bone graft and ultimately implant failure [9,31]. Therefore, to minimize the risk of postoperative complications and ensure surgical success, careful preoperative assessment of ostium patency and anatomical variations that may predispose to sinus pathology using CBCT is of great clinical importance [19]. Studies using CBCT images have been conducted in recent years. Shetty et al. detected concha bullosa using CBCT images and pre-trained models [32]. Esmaeilyfard et al. performed a study detecting cystic lesions. CNN architectures were preferred in this study [33]. Yang et al. performed maxillary sinus segmentation and bone graft analysis using CBCT images and a 2D U-Net architecture in their study [34]. This study aimed to detect ostium opening from CBCT images. Artificial intelligence-based systems were used to ensure the highest reliability in the assessment process. To further improve the success of the analysis, hybrid models with higher performance were preferred over standard AI architectures, and the performance differences between these and other architectures were examined.

The main reason the proposed model performed better than other architectures is the combined use of complementary features from different deep learning architectures. The ConvNeXt-Tiny architecture, due to its convolution-based structure, effectively learns local and low-level features in images. In contrast, the DeiT model, through its transformer-based architecture, can model broader contextual and global relationships. Combining features from these two architectures increases the model’s local and global representational power, enabling it to learn more distinctive features. Furthermore, the gated MLP-based feature fusion mechanism in the proposed model dynamically regulates information flow among features from different backbone models, thereby highlighting more important features. This mechanism allows the model to suppress unnecessary or low-contributing features while assigning greater weight to more distinctive ones. This contributes to improved classification performance, especially when similar patterns exist between classes. In conclusion, an examination of the performance metrics reveals that the proposed model aims to function as a decision-support tool, rather than replace clinical experts. The proposed model can assist clinicians by rapidly screening CBCT scans and prioritizing cases suspected of having ostium-related abnormalities, thereby reducing the diagnostic workload. The proposed model for ostium detection may be particularly useful in clinical settings where early diagnosis and workflow efficiency are critical. However, our study also has some limitations. Since the dataset used in this study was collected from a single center, it was not possible to test how well the model performs with data from different regions. The small number of patients used in the study and the imbalance between the classes are other limitations. In our future studies, we aim to conduct research using multi-center data.

## 5. Conclusions

This study developed a hybrid deep learning model that automatically detects the status (open or closed) of the maxillary sinus ostium to prevent complications in maxillary sinus lift and implant surgeries. The aim was to use artificial intelligence to improve the reliability of the assessment process, which is currently performed manually by physicians during clinical examinations and is therefore time-consuming and open to interpretation. To this end, the detail-capturing capabilities of the CNN-based ConvNeXt-Tiny architecture were combined with the transformer-based DeiT architecture, and this structure was enhanced with a gated MLP fusion mechanism. The developed hybrid model was compared with six different artificial intelligence architectures commonly used in the literature. According to the experimental results, our proposed model achieved the highest success rate compared to individual models, with a test accuracy of 95.03%, an F1 score of 94.18%, and a very low error rate of 4.96%. The results demonstrate that the gated fusion mechanism statistically significantly improves classification performance by suppressing unnecessary features and highlighting distinctive details. In conclusion, this innovative model provides clinicians with a highly accurate, fast, and objective decision-support tool for detecting ostium patency in radiographic images. The use of this system in dentistry is expected to reduce human-induced diagnostic errors and complications such as postoperative sinusitis.

## Figures and Tables

**Table 1:** Performance measurement metrics for pre-trained models.

Models	Acc.	Pre.	Recall	F1-Score	NPV	MCC	Error Rate
ResNet50	86.52	87.97	80.65	83.02	90.62	63.26	13.47
DenseNet121	89.36	87.39	88.64	87.96	87.39	77.95	10.63
ConvNeXt-Tiny	89.36	88.79	86.28	87.36	88.79	76.80	10.63
ViT-B/16	83.68	82.65	78.57	80.07	82.65	63.06	16.31
DeiT	87.94	86.00	86.42	86.21	86.00	72.57	12.05
Swin	86.52	84.39	84.79	84.58	84.39	69.56	13.47

**Table 2:** Performance metrics of the proposed model.

Models	Acc.	Pre.	Recall	F1-Score	NPV	MCC	Error Rate
ConvNeXt-Tiny	89.36	88.79	86.28	87.36	88.79	76.80	10.63
DeiT	87.94	86.00	86.42	86.21	86.00	72.57	12.05
ConvNeXt-Tiny + DeiT MLP	90.07	90.65	86.21	87.95	90.65	79.12	09.92
Proposed Model (ConvNeXt-Tiny + DeiT Gated MLP)	95.03	95.08	93.39	94.18	95.08	89.67	04.96

## Data Availability

The original contributions presented in this study are included in the article. Further inquiries can be directed to the corresponding author.
